# An Early Diagnostic Tool for Diabetic Peripheral Neuropathy in Rats

**DOI:** 10.1371/journal.pone.0126892

**Published:** 2015-05-18

**Authors:** Shoista Kambiz, Johan W. van Neck, Saniye G. Cosgun, Marit H. N. van Velzen, Joop A. M. J. L. Janssen, Naim Avazverdi, Steven E. R. Hovius, Erik T. Walbeehm

**Affiliations:** 1 Dept. of Plastic, Reconstructive and Hand Surgery, Erasmus University Medical Center, Rotterdam, The Netherlands; 2 Dept. of Neuroscience, Erasmus University Medical Center, Rotterdam, The Netherlands; 3 Dept. of Anesthesiology, Laboratory of Experimental Anesthesiology, Erasmus University Medical Center, Rotterdam, The Netherlands; 4 Dept. of Internal Medicine, Erasmus University Medical Center, Rotterdam, The Netherlands; San Raffaele Hospital, ITALY

## Abstract

The skin’s rewarming rate of diabetic patients is used as a diagnostic tool for early diagnosis of diabetic neuropathy. At present, the relationship between microvascular changes in the skin and diabetic neuropathy is unclear in streptozotocin (STZ) diabetic rats. The aim of this study was to investigate whether the skin rewarming rate in diabetic rats is related to microvascular changes and whether this is accompanied by changes observed in classical diagnostic methods for diabetic peripheral neuropathy. Computer-assisted infrared thermography was used to assess the rewarming rate after cold exposure on the plantar skin of STZ diabetic rats’ hind paws. Peripheral neuropathy was determined by the density of intra-epidermal nerve fibers (IENFs), mechanical sensitivity, and electrophysiological recordings. Data were obtained in diabetic rats at four, six, and eight weeks after the induction of diabetes and in controls. Four weeks after the induction of diabetes, a delayed rewarming rate, decreased skin blood flow and decreased density of IENFs were observed. However, the mechanical hyposensitivity and decreased motor nerve conduction velocity (MNCV) developed 6 and 8 weeks after the induction of diabetes. Our study shows that the skin rewarming rate is related to microvascular changes in diabetic rats. Moreover, the skin rewarming rate is a non-invasive method that provides more information for an earlier diagnosis of peripheral neuropathy than the classical monofilament test and MNCV in STZ induced diabetic rats.

## Introduction

Diabetic peripheral neuropathy is a common complication of diabetes. Assessment of the density of IENFs in a skin biopsy is considered to be a reliable method to measure diabetic peripheral neuropathy in both human diabetes and animal models with diabetes [1, 2]. However, taking skin biopsies may be considered a risk factor in a disease where wound healing is prone to be disturbed. Moreover, skin biopsies cover only a small area and are invasive. In contrast, corneal nerve parameters and nerve conduction velocity are non-invasive diagnostic tools to determine diabetic peripheral neuropathy. However, corneal nerve parameters do not directly test the areas that are affected by peripheral neuropathy. Furthermore, nerve conduction is a measure of the degree to which an axon is myelinated and therefore neglects the small unmyelinated fibers [3].

Reduction of nerve conduction velocity in diabetic peripheral neuropathy has been shown to be preceded by impaired vasodilation of the arterioles [4]. Consistent with this, thermography studies have reported decreased skin temperatures in diabetic subjects with microvascular disease before other clinical signs of peripheral neuropathy can be identified [5–7]. In addition, the rewarming rate of the skin after cooling has been demonstrated to be useful for early diagnosis of diabetic peripheral neuropathy [8, 9]. Taken together, these data suggest that signs of microvascular disease are already present prior to the development of clinically overt diabetic peripheral neuropathy. Therefore, monitoring changes in skin rewarming rate may enable early diagnosis and management of diabetic peripheral neuropathy, leading to reduction in the development of foot ulcers [10] and infections, which are often followed by amputations.

The STZ induced diabetic rat is one of the most frequently used animal models to study diabetes [11, 12]. However, the exact relationships between microvascular changes and diabetic peripheral neuropathy have not been studied in this animal model.

Therefore, in the present study, microvascular changes in the plantar skin of STZ diabetic rats were studied and related to the development of diabetic peripheral neuropathy. In addition, the onset of detectable changes by classical diagnostic tools such as sensitivity to the intensity of pressure, the density of IENFs and MNCV were compared to the skin rewarming rate in STZ diabetic rats. With these tools it was investigated whether the skin rewarming rate could serve as an early diagnostic tool for diabetic peripheral neuropathy in STZ diabetic rats as is shown in human diabetes.

## Materials and Methods

### Animals

WAG/RijHsd female rats (n = 27, 10 weeks old, weighing 130–150 gram) were purchased from Charles River (l'Arbresle, France)[13]. The animals were pair-housed in hooded cages at room temperature on a 12-hour light/dark schedule, and were given water and food ad libitum. All experiments were approved by the Dutch Ethical Committee on Animal Welfare according to the European guidelines for the care and use of laboratory animals (Council Directive 86/6009/EEC).

### Induction of diabetes

Diabetes was induced in 21 rats by a single intra-peritoneal injection of STZ (Sigma-Aldrich, St. Louis, MO, USA) at a dose of 65 mg/kg body weight in 0.05 mol/L sodium citrate buffer, pH 4.5, as described previously [13]. The rats were randomly assigned into 3 groups: A, B and C (n = 7 in each group). Following diabetes induction, group A was killed after 4 weeks, group B after 6 weeks and group C after 8 weeks. The control group consisted of 6 rats who received a single intra-peritoneal injection with an equal volume of vehicle without STZ. Control rats were followed for 8 weeks. Blood glucose was measured from tail vein blood by a glucometer (OneTouch, LifeScan, Milpitas, California, USA). Diabetes was diagnosed in rats, when blood glucose levels were higher than 20 mmol/L during the entire 4 weeks after the induction of diabetes. Insulin treatment was not given.

### The blood flow and oxygenation of the plantar hind paws’ skin

A combined laser doppler flowmetry and spectrophotometry system (O2C, LEA Medizintechnik, Giessen, Germany), which has been applied in human and animal studies [14, 15], was used to non-invasively measure blood flow and oxygen saturation of the glabrous plantar hind paws. In both diabetic and control rats the percentage oxygen saturation and amount of skin blood flow were assessed at 4, 6, and 8 weeks.

### Rewarming rate after cold exposure

The temperature of the skin was assessed using the built-in infrared digital video camera (320 × 240 pixels) by 1 Hz data acquisition system (ThermaCAM Researcher 2001 HS; FLIR Systems, Berchem, Belgium), and all data were continuously collected by a laptop. The distance between the camera and the hind paw was 13 cm ± 2 cm. The pixel size of the temperature recordings was 0.8 × 0.8 mm. The skin temperature of the entire plantar hind paw was recorded while the animal was fixed after placing the animal on a 14°C plate for 5 seconds. The minimum temperature of the plantar hind paws were exported to text files using ThermaCAM Researcher Pro (version 2001-HS; FLIR Systems, Wilsonville, Oregon, USA). The area of interest was selected by drawing a line surrounding the entire plantar hind paws. The average rewarming rate is demonstrated as the increase in skin temperature per 120 seconds.

### Thermal sensitivity

In order to determine the occurrence of thermal hypersensitivity, cold and hot plate tests were performed as described previously [16, 17]. In short, rats were placed in an open-ended chamber with clear walls with a surface temperature of either 5°C (cold plate) or 50°C (hot plate). These experiments were performed on separate days to prevent interference. The time until hind paw withdrawal or licking was observed.

### Von Frey test

In the von Frey test, used to determine the mechanical sensitivity threshold for nociception, each rat was placed in a chamber with a mesh metal floor. Then, von Frey hairs, ranging from 2 to 300 grams, were applied 5 times, and was scored positive when a minimum of 3 paw flicks (the animal's reflex withdrawal response) were observed, as described previously [18]. The control group served as the reference group.

### Electromyography (EMG)

Innervation of motor axons in muscles was evaluated by recording the evoked CMAP peak-peak amplitudes and latencies of the gastrocnemius muscles [19] in the diabetic groups and control animals as described previously [20]. CMAP peak-peak amplitudes and latencies were recorded and averaged over a batch of 20 responses. The average amplitudes in each diabetes group were compared to the control group. The MNCV was calculated as the distance of stimulating electrode to recording electrode (mm)/latency (ms).

### Tissue preparation

After 4, 6, or 8 weeks, the animals were killed by an overdose of pentobarbital (100mg/kg ip). For each rat, the plantar skin of the hind paw was dissected and directly immersion-fixed in 2% paraformaldehyde-lysin-periodate (PLP) for 24 hours at 4°C. The skin was further processed and embedded in gelatin as described previously [21]. Finally, the embedded skin was sectioned at 40 μm with a freezing microtome and collected in glycerol for long-term storage at -20°C.

The pancreas tissue of the rats was harvested, fixed in 10% neutral buffered formalin solution, and embedded in paraffin. Subsequently, these specimens were stained with hematoxylin and eosin (H&E). Each specimen was evaluated by a bright-field microscope and scanned into digital slides (Nanozoomer 2.0 series, Hamamatzu, Japan).

### Immunohistochemistry

Immunohistochemistry of the skin sections was performed as previously described to semi quantify the density of sensory nerve fibers innervating the skin [21], and to evaluate the presence of CD-31 positive endothelial cells. The skin sections were incubated for 48 hours in a cocktail of 2% BSA containing the diluted primary antibody Protein Gene Product 9.5 (PGP9.5, 1/10.000, anti-rabbit, Enzo Life Sciences, New York, USA), or anti-CD31^+^ (1/5000, anti-rabbit, Spring Bioscience, California, USA) at 4°C. Subsequently, skin sections where incubated with the appropriate secondary biotinylated antibody labeled with horseradish peroxidase (HRP) (1/200, Biotine, Sigma-Aldrich, St. Louis, MO, USA) for 90 min at RT. The 3, -3’ diaminobenzidine (DAB) reaction was then used to reveal the antigenic binding sites of the primary antibodies [22]. Thereafter, the sections were mounted on slides and the CD31^+^ stained sections stained with 0.05% thionin for 4 minutes, which colored the keratinocytes blue. Finally, all skin sections were dehydrated using absolute ethanol (< 0.01% methanol), transferred to xylene, and cover slipped with Permount (Fisher Scientific, Hampton, NH).

Each skin section was scanned in 3 layers of 8μm each by Nanozoomer 2.0 series (Nanozoomer 2.0 series, Hamamatzu, Japan). Four proximal and 4 distal skin sections of the plantar hind paw were quantified for epidermal nerve fibers in the center part of the plantar hind paw (80.000 μm^2^) using a 40x objective in ImageScope software (Aperio ImageScope v11.1.2.760) [23, 24]. The average labeled nerve fibers per mm^2^ and the average epidermal thickness were calculated for each rat. Percentage CD31-positive cells was calculated by Leica Cell-D (Olympus, Imaging software for life science microscopy, USA) in 4 proximal and 4 distal skin sections over the entire upper dermis of the plantar hind paw.

### Statistical analysis

Results were presented as means with standard error of the mean (SEM). An one-way ANOVA with Tukey post hoc test was performed to determine the differences between the experimental groups. In addition, a two-way analysis of variance (ANOVA) with one repeated-measures factor time, with a Bonferroni post-test, was used to determine the overall differences when the test was performed at different examined time points in the same group. The average skin rewarming rate was compared between the diabetic rats and controls at different time intervals using an unpaired Students’ t-test. A *p*-value of 0.05 or less was considered statistically significant. Data were analyzed using Graph Pad Prism software (GraphPad Prism Inc., San Diego, California, USA) version 5.0b for apple MacBook.

## Results

**Fig 1:**
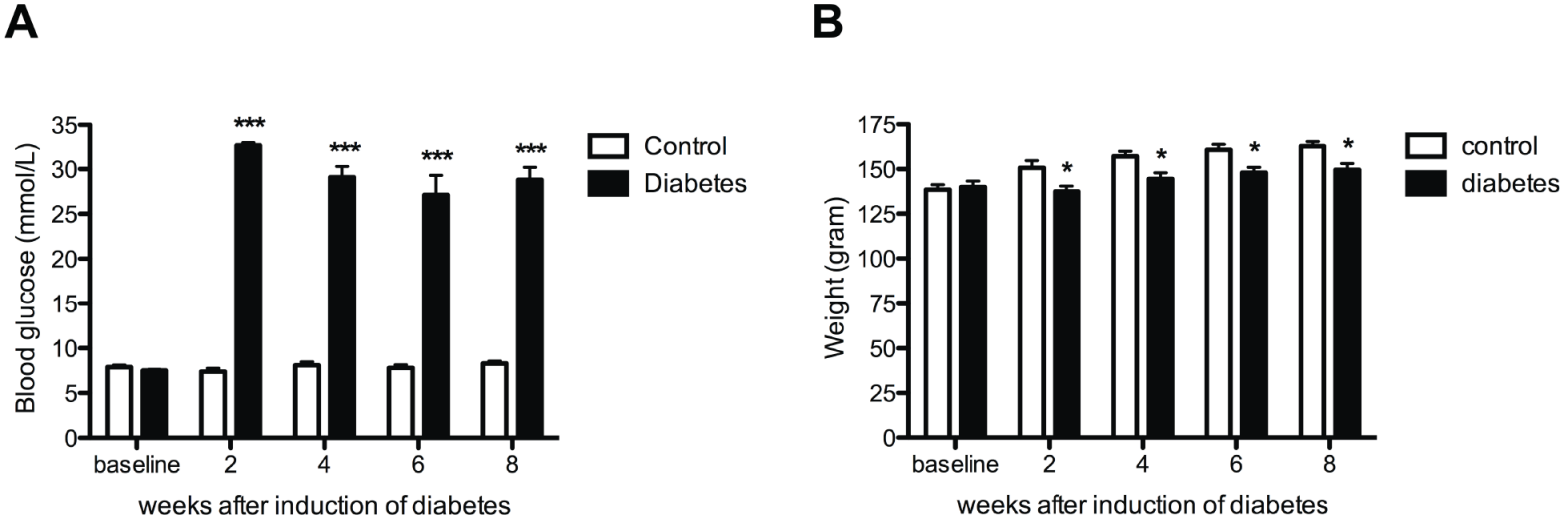
Increased blood glucose and stable body weight in diabetic animals. Blood glucose was increased in all diabetic animals (black bars) when compared to controls (white bars) (**A**). Significantly smaller increase in body weight is demonstrated in the diabetic animals (black bars) when compared to controls (white bars) (**B**). Data are presented as mean ± SEM. *p <0.05, ***p <0.001 (One-way ANOVA with Tukey post hoc test).

Diabetic animals showed more than three times higher blood glucose levels than control animals during the whole study period (Fig 1a). One week after induction of diabetes by STZ, all rats demonstrated polydipsia and polyuria (data not shown). The body weight of the diabetic rats was significantly less compared to controls at all experimental time points studied but did not significantly change across time points (Fig 1b).

**Fig 2:**
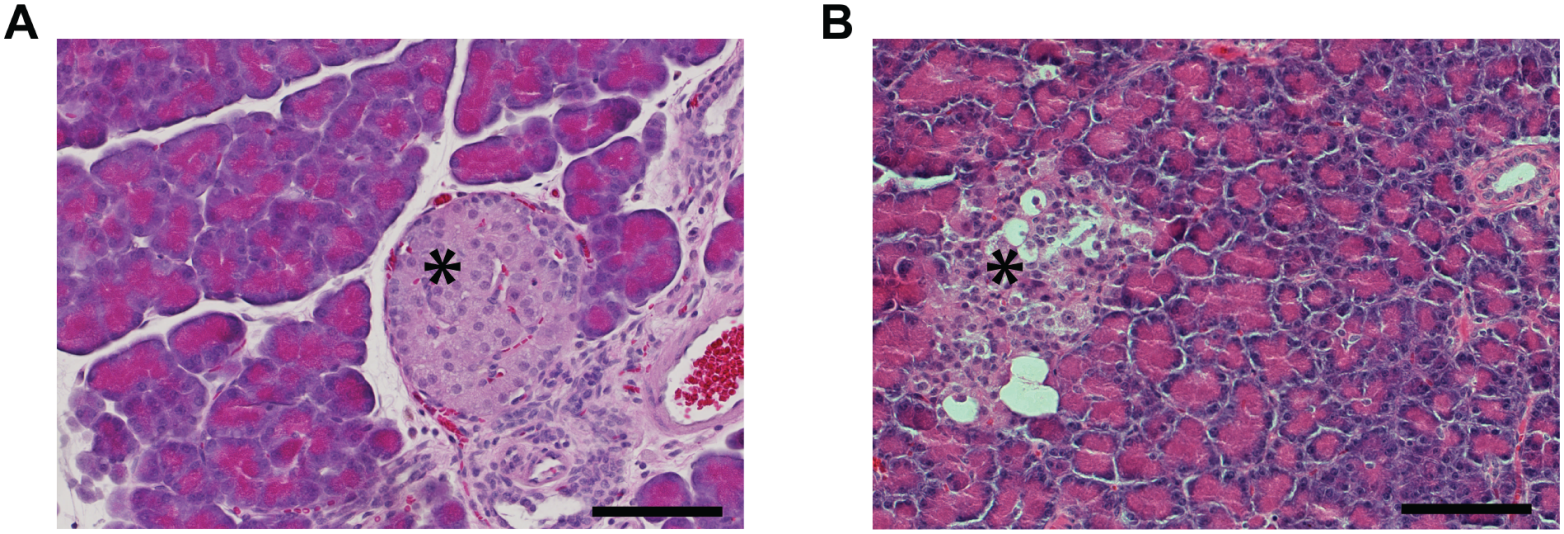
Degeneration of pancreatic tissue in diabetic animals. Hematoxylin and eosin stained Fig.s of the pancreas (**A, B**) showing that the cells and the Islets of Langerhans were considerably smaller in the diabetic pancreas (**B**) compared to control (**A**). Scale bar 100um.

Eight weeks after induction of diabetes, no pathological changes were observed in the pancreatic tissue of the control group (Fig 2a). However, in the diabetic group the Islets of Langerhans showed shrinkage and displayed degenerative and necrotic changes due to the toxic effects of STZ already 4 weeks after induction of diabetes (Fig 2b).

### Microvascular changes in diabetic rats

**Fig 3:**
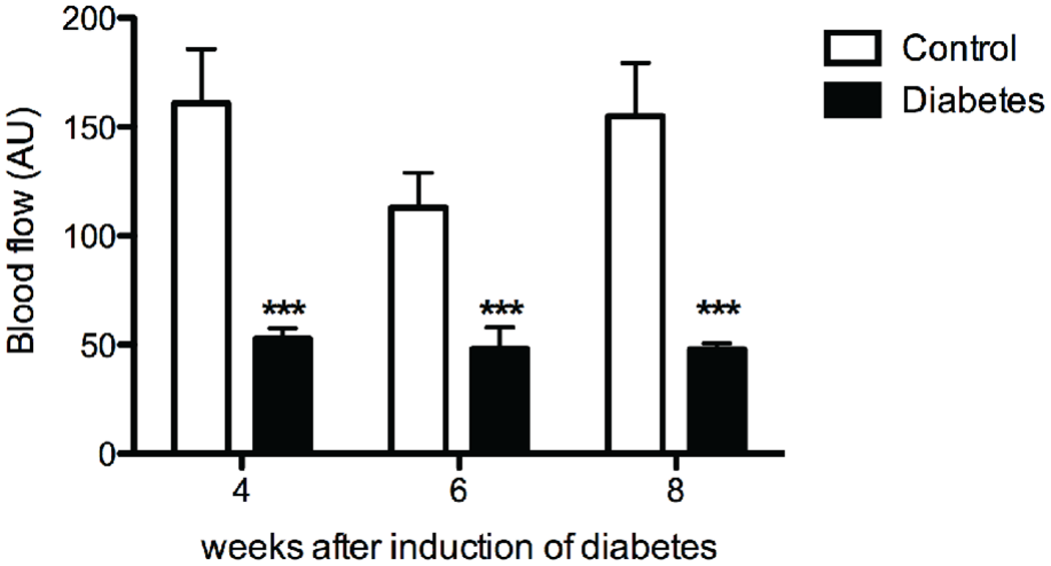
Decreased skin blood flow in diabetic animals. Plantar skin blood flow was decreased in all diabetic animals (black bars) when compared to controls (white bars). Data is presented as mean ± SEM. *** p< 0.001 (two-way ANOVA with one repeated-measures factor ‘time’ with Bonferroni post-test).

**Fig 4:**
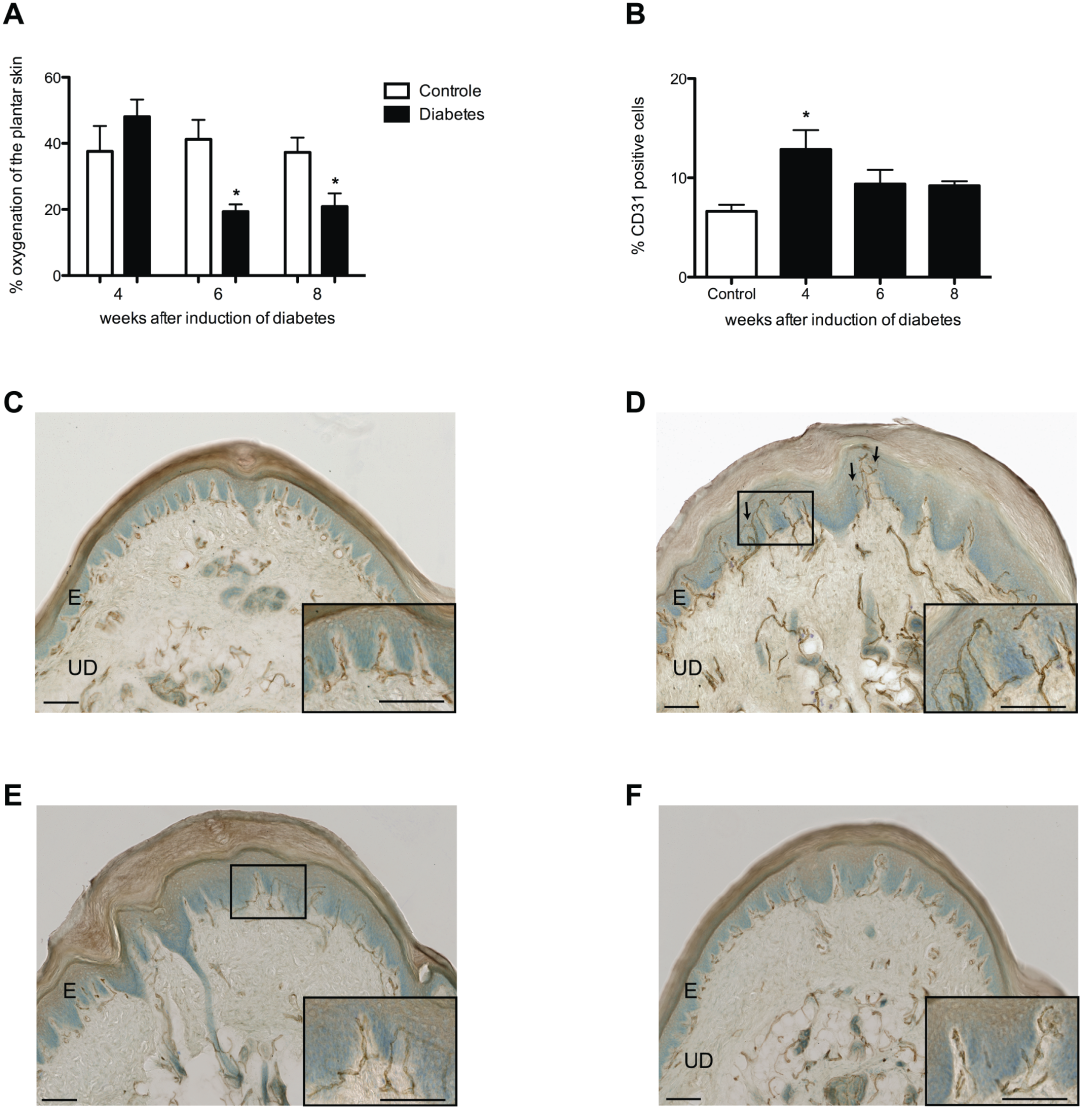
Percentage skin oxygenation and CD31-positive cells in the plantar skin. Decreased percentage in skin oxygenation was observed in diabetic animals (black bars) 6 and 8 weeks after the induction of diabetes when compared to controls (white bars) (**A**). Four weeks after induction of diabetes (black bar) a significantly increased percentage CD31-positive cells was observed in diabetic rats when compared to controls (white bar) (**B**), which is illustrated in histological scans of controls (**C**), 4 (**D**), 6 (**E**), and 8 (**F**) weeks after the induction of diabetes. Arrows indicate sprouting angiogenesis. Scale bars 50um.

Four weeks after the induction of diabetes, the blood flow in the skin of the plantar hind paw was significantly lower in diabetic rats than in controls (Control 4 weeks: 160.8 ± 61.2 AU vs. 4 weeks diabetes: 52.7 ± 11.8 AU; *p*<0.001) (Fig 3). In contrast, the percentage of CD31-positive endothelial cells in the glabrous skin of the hind paw was significantly higher 4 weeks after induction in diabetic rats than in controls (Control: 6.6 ± 0.7% vs. 4 weeks diabetes: 12.9 ± 1.9%; *p* < 0.05) (Fig 4a). However, while the blood flow remained low during further follow-up at 6 and 8 weeks after induction of diabetes, no significant difference in the percentage of CD31-positive endothelial cells was seen between diabetic rats and controls at these time points (Fig 4b–4f).

Four weeks after induction of diabetes, skin oxygenation was not different between diabetic rats and controls (Fig 4a). However, 6 weeks after induction the skin oxygenation in diabetic rats became significantly lower than in controls (*p*< 0.05) (Fig 4a).

**Fig 5:**
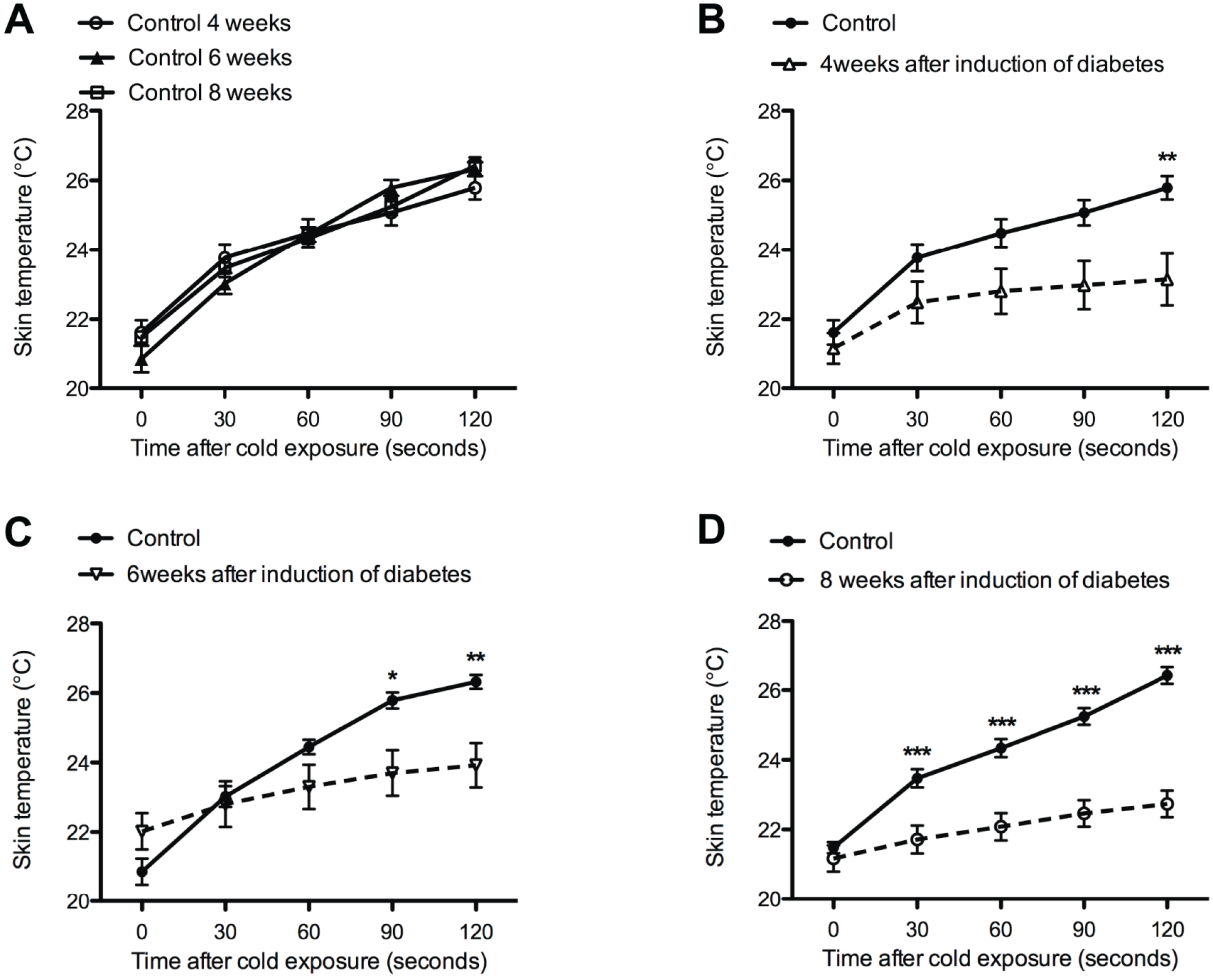
Decreased skin temperature of the plantar hind paw after cold exposure. Control groups were not significantly different (**A**). Significant lower skin temperatures were observed in diabetic animals 4 (**B**), 6 (**C**), and 8 weeks (**D**) after the induction of diabetes (dotted line) when compared to controls (continuous line). Data is presented as mean ± SEM. *p < 0.05, **p <0.01, *** *p*< 0.001 (two-way ANOVA with one repeated-measures factor ‘time’ with Bonferroni post-test).

The skin rewarming rate in the controls were not significantly different at 4, 6 and 8 weeks (Fig 5a). Directly after cooling the skin (t = 0 seconds), no significant differences in skin temperature were observed between the controls and the 3 diabetic groups (Fig 5b–5d). However, the difference in temperature between controls and diabetic rats becomes significant at earlier time points after cooling, 120 seconds after cooling in rats 4 weeks post induction, 90 seconds after cooling in rats 6 weeks post induction, and after 30 seconds in rats 8 weeks post induction. This indicates that there is a progressively bigger delay in rewarming at later time points after induction of diabetes (Table 1).

**Table 1:** Decreased rewarming rate of the plantar skin after cold exposure.

Average increase in temperature per 120 seconds ± SEM (°C)			
Time (weeks)	Control	Diabetes	T-test
4	4,3 ± 0,04	2,0 ± 0,6	*p* < 0,01
6	5,5 ± 0,5	1,9 ± 0,3	*p* < 0,001
8	4,9 ± 0,1	1,6± 0,3	*p* < 0,001

Progressive decrease in the average skin temperature (degree Celsius) per 120 seconds is shown in diabetic animals when compared to control. Data is presented as mean ± SEM (Unpaired T-test).

### Changes in skin innervations in diabetic rats

**Fig 6:**
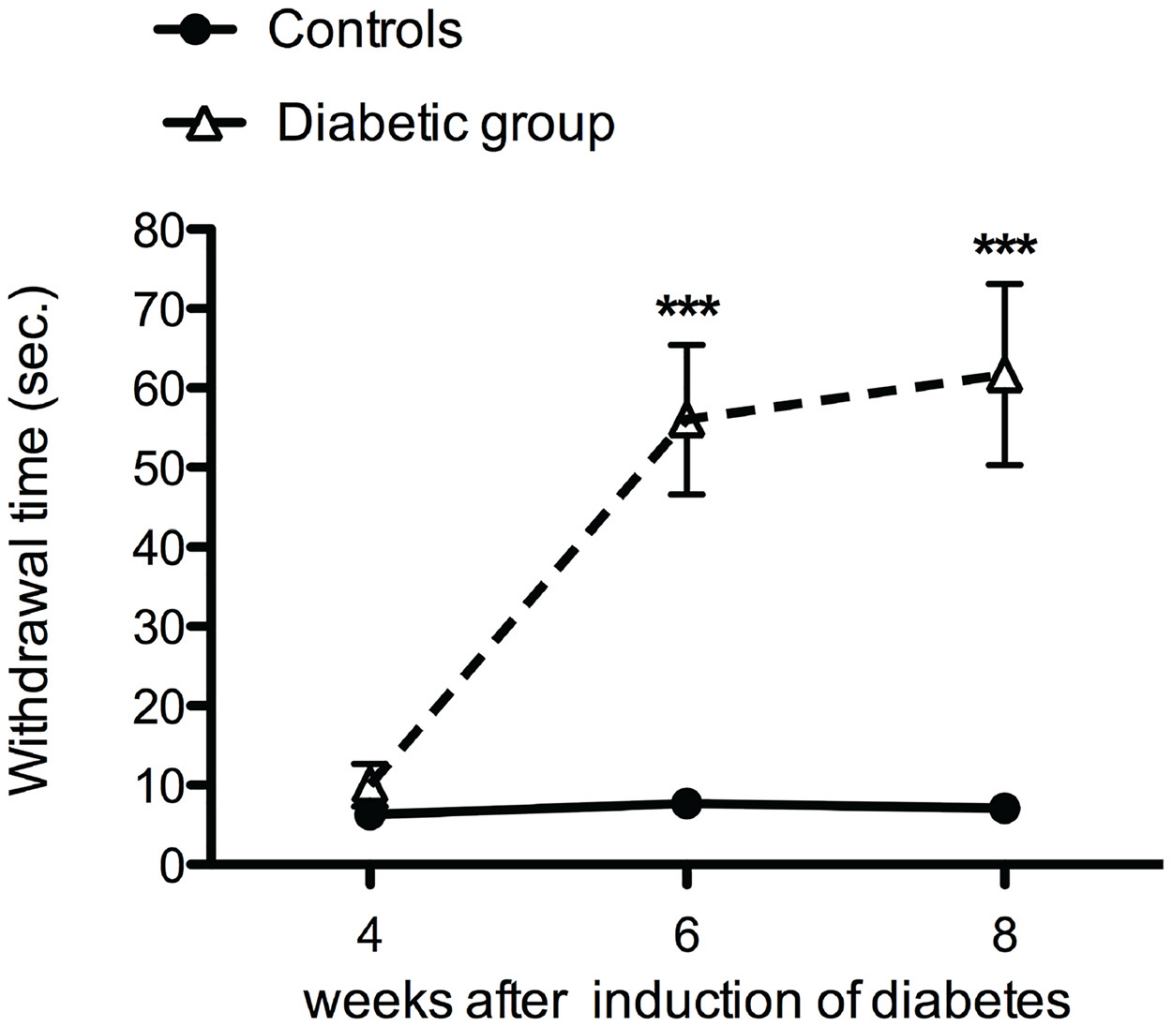
Diabetic animals developed mechanical hyposensitivity. Increased mechanical withdrawal threshold was observed 6 and 8 weeks after induction of diabetes (dotted line) when compared to control (continuous line) Data is presented as mean ± SEM. *** *p*< 0.001 (two-way ANOVA with one repeated-measures factor ‘time’ with Bonferroni post-test).

Four weeks after the induction of diabetes, no significant difference in the mean withdrawal response to mechanical stimuli were observed between the diabetic rats and the control rats (Fig 6). However, 6 and 8 weeks after the induction of diabetes, a significant increase in the mean withdrawal threshold was observed in diabetic rats (Fig 6).

**Fig 7:**
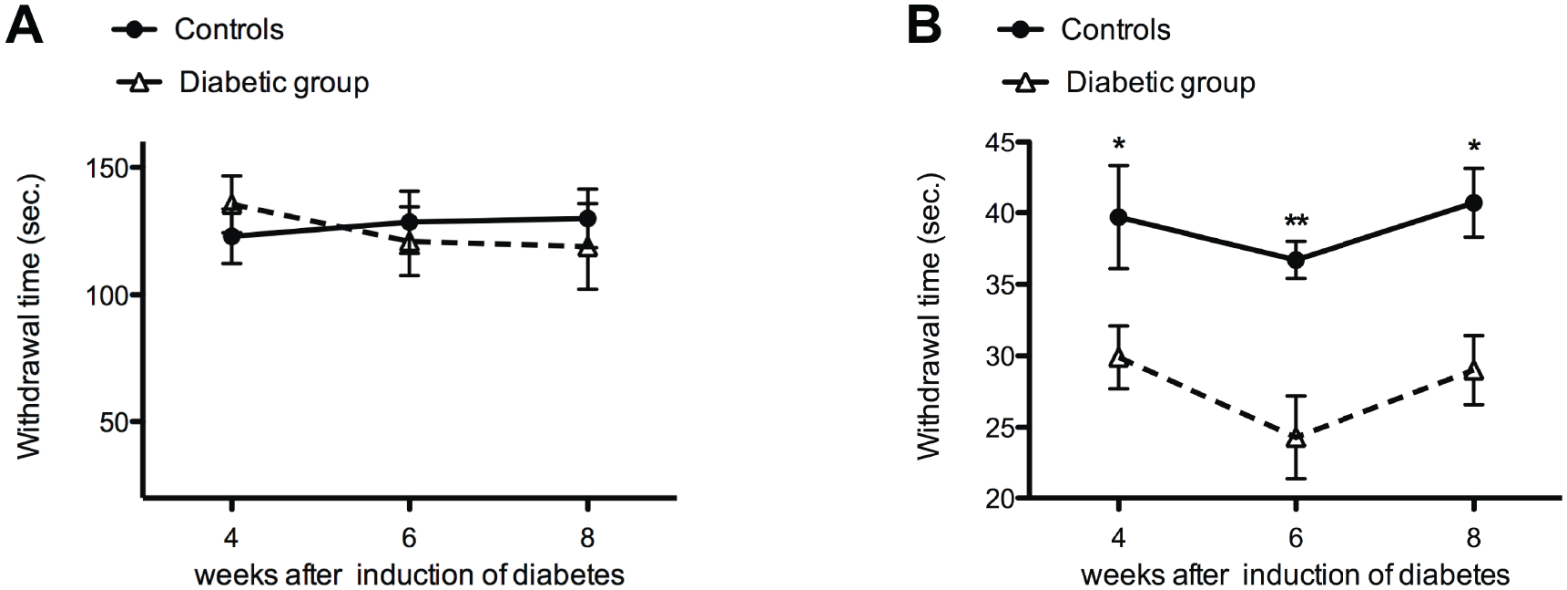
Heat hypersensitivity in diabetic animals. No significant difference was observed between the diabetic animals (dotted line) and controls (continuous line) for the cold plate test (5°C) (**A**). In contrast to the cold plate test, decreased withdrawal time was observed for the hot plate test (50°C) in diabetic animals (dotted line) when compared to control (continuous line) at all experimental time points (**B**).

While the withdrawal latency for cold plate remained similar as control at all examined time points (Fig 7a), a significant decrease was seen in the withdrawal latency for the hot plate test at 4, 6 and 8 weeks after the induction of diabetes suggesting heat hypersensitivity (Fig 7b).

**Fig 8:**
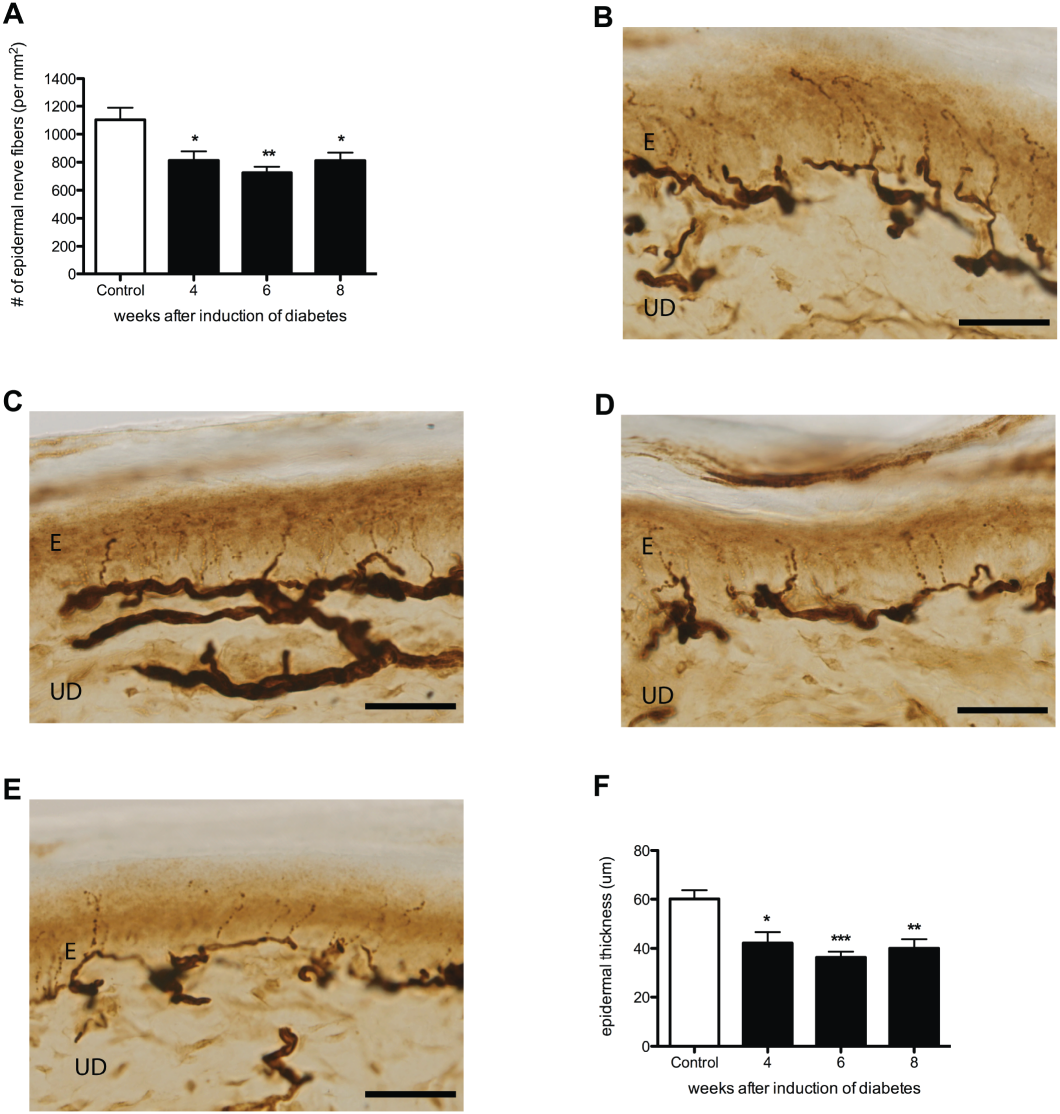
Decreased innervation and decreased epidermal thickness in STZ diabetic rats. Decreased density PGP9.5-IR nerve fibers (**A**) and average epidermal thickness (**F**) was demonstrated in the plantar skin of diabetic animals 4, 6, and 8 weeks after induction of diabetes (black bars) when compared to control (white bar). This is illustrated by histological sections of plantar skin in controls (**B**), and 4 (**C**), 6 (**D**), and 8 (**E**) weeks after induction of diabetes. Data is presented as mean ± SEM. *p < 0.05, **p <0.01 (One-way ANOVA with Tukey post hoc test). E = epidermis, UD = upper dermis, scale bar = 100um.

**Fig 9:**
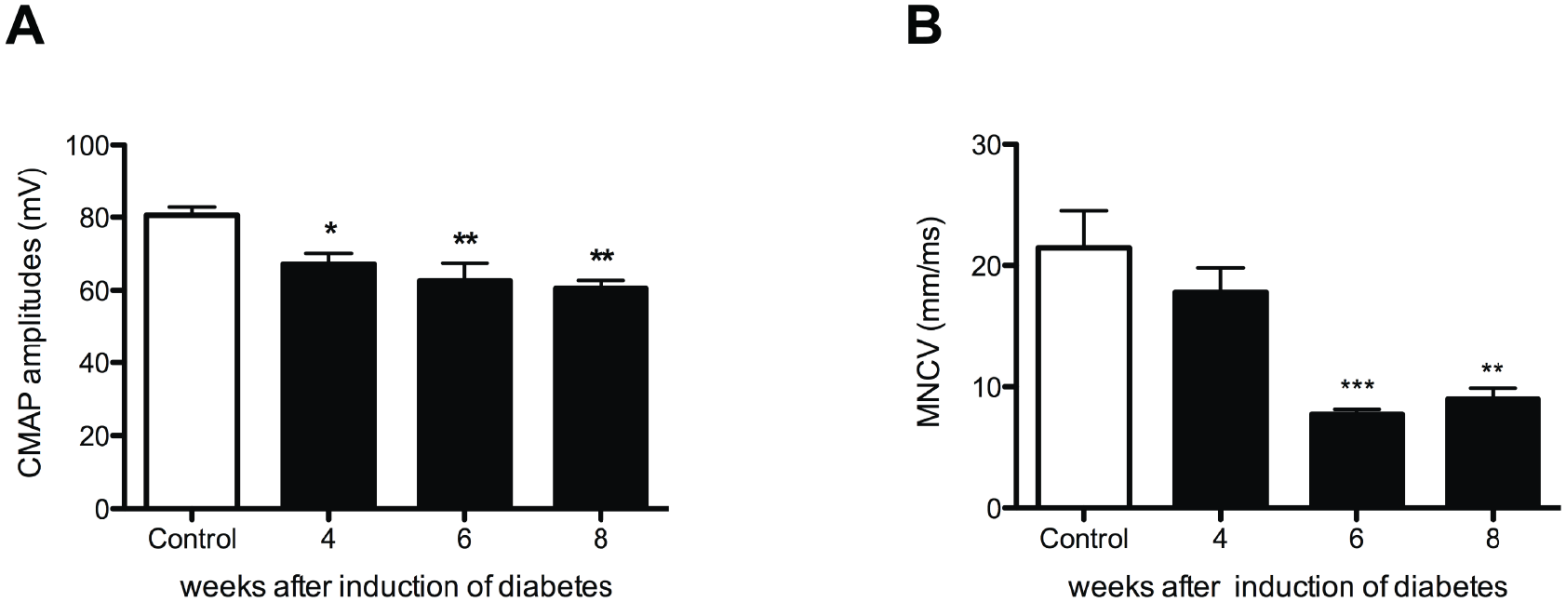
Electrophysiological changes in diabetic animals. CMAP amplitude decreased in all diabetic groups (black bars) when compared to controls (white bars) (**A**), while MNVC showed a significant decrease 6 and 8 weeks after induction of diabetes (**B**).

IENFs were stained by the pan-neuronal protein gene product 9.5 (PGP9.5) marker. The average density of PGP9.5-IR nerve endings in the plantar skin was significantly lower in diabetic rats than in controls from 4 weeks onward (Fig 8b–8d). This decreased skin innervation was accompanied by a significantly decreased epidermal thickness compared to controls (Fig 8a). Similar to the density of PGP9.5-IR nerve endings, significant decrease was observed in the average amplitude of evoked gastrocnemius muscle CMAPs in all diabetic groups (Control: 80.5 ± 2.2 mV vs. 4 weeks diabetes: 67.2 ± 2.7 mV; *p*<0.05, vs. 6 weeks diabetes: 62.6 ± 4.5 mV; *p*<0.01, and vs. 8 weeks diabetes: 60.5 ± 2.1 mV; *p*<0.01)(Fig 9a). However, the decrease in MNCV was seen later, 6 and 8 weeks after the induction of diabetes suggesting demyelination of motor nerves (Fig 9b).

## Discussion

The aim of our study was to investigate if the skin rewarming rate could serve as an early diagnostic tool for diabetic peripheral neuropathy in STZ diabetic rats as is shown in human diabetes [8]. Although the STZ diabetic rat model has been used to study complications of diabetes for many years [25–27], there is no information available concerning the rewarming rate of the skin and its correlation to diabetic peripheral neuropathy in this model. In the present study, we quantified microvascular changes in the plantar skin of the rats’ hind paw after the induction of diabetes and investigated whether these microvascular changes were associated with (the development of) diabetic peripheral neuropathy.

STZ is known to have toxic effects on beta cells in the Islets of Langerhans [28–30]. In the current study, besides increased blood glucose, morphological changes were observed in the pancreatic tissue of the STZ diabetic rats confirming a normal progression of disease in our experimental animals.

### Microvascular changes in diabetic rats

A decrease in the skin blood flow of the plantar hind paw was observed after the induction of diabetes at all measured time points. Analogous observations have been reported previously [31]. However, in human diabetes, it has been found that early in the development of diabetic microvascular complications there is initially an increase in the skin blood flow before the blood flow decreases during progression of disease [32]. Therefore, we performed additional blood flow measurements at 2 and 3 weeks after the induction of diabetes. Nevertheless, no significant difference was found in blood flow measurements of 2 and 3 weeks diabetic animals when compared to controls.

Interestingly, despite the decreased skin blood flow, initially no significant changes were seen in the skin oxygenation 4 weeks after the induction of diabetes. This finding suggests that the rats’ circulation system maintained a normal oxygenation of the skin by a relative increase of CD31-positive endothelial cells in the upper dermis 4 weeks after the induction of diabetes. Although hypoxia is associated with angiogenesis, we were able to show a rise in CD31-positive cells as a response to the decrease in blood flow when oxygenation was comparable to control values [33]. However, 6 and 8 weeks after the induction of diabetes, the relative significant increase in CD31-positive cells was no longer present, while the skin blood flow remained decreased, which resulted in hypoxia of the plantar skin. These findings suggest that the body was no longer able to compensate for the decreased skin blood flow from 6 weeks after the induction of diabetes.

The decreased skin blood flow was accompanied by a significant delay in the rewarming rate of the skin in diabetic rats compared to controls. These findings in STZ diabetic rats support the direct correlation between skin temperature and skin blood flow as is shown in human diabetes [34]. Moreover, our diabetic animal model resembles skin rewarming rate of human diabetes. However, in human diabetes, a significant difference in the skin temperature was not observed until 10 minutes after cooling. In our study, significant differences in rewarming rate between the diabetic rats and the controls were already found after 120 seconds of monitoring. While human thermography studies use exposure to 14°C cold water, we observed in a pilot study that placement of STZ diabetic rats on a 14°C cold plate for 40 seconds was sufficient to detect differences in rewarming rate after 120 seconds (data not shown). Cold water exposure and longer monitoring of the plantar skin would have required administration of anesthesia, which is shown to have an effect on the blood flow and skin temperature and therefore was avoided in our study [35, 36].

### Changes in skin innervations in diabetic rats

Four weeks after the induction of diabetes, no significant changes were observed in mechanical sensitivity, while more than 30% IENF loss was observed. These results demonstrate, similar to human diabetes that less innervation of the skin does not immediately lead to a measurable decrease in mechanical sensation [1]. However, further decline in IENFs resulted in mechanical hyposensitivity 6 weeks after the induction of diabetes [37]. From this finding we suggest that a threshold of the density of IENFs needs to be reached in order to cause changes in mechanical sensitivity: a similar phenomenon was also shown after peripheral nerve injury in rats [38]. In addition, decreased density of IENFs in the plantar skin of diabetic rats was accompanied by a significantly decreased epidermal thickness. Decrease in epidermal thickness of the plantar foot is also observed in human diabetic neuropathy when compared to healthy controls [39] supporting the important role of IENFs in proliferation of keratinocytes in the epidermis [40].

In contrast to decreased sensitivity to mechanical stimuli, the diabetic animals demonstrated hypersensitivity to heat. This finding supports the previously suggested separate pathways for conducting mechanical and thermal information [41]. However, further research identifying the subgroup of IENFs is required to confirm the modality-specific contribution of these sensory nerve fibers in diabetic peripheral neuropathy. Moreover, while hypersensitivity was observed for heat (50°C), no significant changes were observed for cold (5°C) temperature. This temperature-induced difference in hypersensitivity may be caused by changes in the expression of different thermo-sensitive transient receptor potential (TRP) channels expressed on IENFs that become activated at >42°C (TRPV1 channel) and <17°C (TRPA1 channel) involved in temperature signaling [42].

The CMAP amplitude in the present study decreased prior to slowing of MNCV demonstrating the appearance of independent axonal degeneration followed by demyelination, which is in line with previous findings from electrophysiological studies performed in human diabetes and as well as in animal models of diabetes [3, 43].

The CMAP amplitude was significantly decreased 4 weeks after induction of diabetes, a time point at which no changes were observed in mechanical sensitivity. This is consistent with findings in human diabetes, demonstrating that subclinical peripheral neuropathy can be early detected by electrophysiological tests [44, 45]. Based on our results we conclude that the skin rewarming rate detects diabetic peripheral neuropathy in STZ diabetic rats, when loss of sensation measured by monofilaments is not yet present. Therefore, our study supports the use of the skin rewarming rate as an early diagnostic tool for asymptomatic diabetic peripheral neuropathy in STZ diabetic rats.

## Supporting Information

S1 Dataset (XLSX)
