# Correction: An Early Diagnostic Tool for Diabetic Peripheral Neuropathy in Rats

**DOI:** 10.1371/journal.pone.0131144

**Published:** 2015-06-15

**Authors:** Shoista Kambiz, Johan W. van Neck, Saniye G. Cosgun, Marit H. N. van Velzen, Joop A. M. J. L. Janssen, Naim Avazverdi, Steven E. R. Hovius, Erik T. Walbeehm

The affiliation for the eighth author is incorrect. The correct affiliation for Erik T. Walbeehm is Dept. of Plastic Surgery, Radboud UMC, Nijmegen, The Netherlands.
